# Cribriform adenocarcinoma of the minor salivary glands: a case report

**DOI:** 10.1186/s13256-023-03875-4

**Published:** 2023-04-18

**Authors:** Tanya Chen, Mohammed Mamdani, Allan Vescan, Christina MacMillan, Joel Davies

**Affiliations:** 1 Department of Otolaryngology - Head & Neck Surgery, Mount Sinai Hospital, 600 University Avenue, Suite 401, Toronto, ON M5G 1X5 Canada; 2 Department of Laboratory Medicine & Pathobiology - Anatomic Pathology, Mount Sinai Hospital, Toronto, ON Canada

**Keywords:** Papillary thyroid cancer, Polymorphous adenocarcinoma, Base of tongue

## Abstract

**Background:**

Polymorphous adenocarcinoma is the third most common malignant salivary gland tumor. Within polymorphous adenocarcinoma, cribriform adenocarcinoma of salivary glands is a rare subtype and resembles papillary thyroid carcinoma histopathologically. Diagnostically, cribriform adenocarcinoma of salivary glands is challenging for pathologists and surgeons alike as initial presentation and cytologic nuclear features can be easily confused with papillary thyroid carcinoma arising from a thyroglossal duct remnant or lingual thyroid.

**Case presentation:**

A healthy 64-year-old Caucasian woman presented to a community otolaryngologist with a 4-year history of progressive postnasal drip, globus sensation, and eventual dysphonia. Flexible fiberoptic laryngoscopy showed a large, smooth, vallecular lesion filling the oropharynx. Computed tomography imaging of the neck showed a rounded heterogeneous mass centered within the right aspect of the oropharynx measuring 4.2 × 4.4 × 4.5 cm. Fine needle aspiration biopsy was suspicious for papillary carcinoma due to microscopic findings of malignant cells, nuclear grooves, and a powdery chromatin pattern. In the operating room, the tumor was resected en bloc using a lateral pharyngotomy approach with partial resection of the right lateral hyoid. A limited cervical lymphadenectomy was performed to facilitate the lateral pharyngotomy approach and two out of three lymph nodes demonstrated regional metastatic disease. Nuclear grooves, nuclear membrane notching, and occasional intranuclear pseudoinclusions were identified, which are overlapping histopathological characteristics of papillary thyroid carcinoma and cribriform adenocarcinoma of salivary glands. It was negative for thyroglobulin and thyroid transcription factor-1, which was in keeping with cribriform adenocarcinoma of salivary glands rather than papillary thyroid carcinoma.

**Conclusion:**

It is difficult to distinguish cribriform adenocarcinoma of salivary glands from papillary thyroid carcinoma solely by cytology, and the distinct characteristics of regional lymph node metastasis coupled with nuanced histologic differences should be emphasized in the evaluation of patients presenting with neck lymphadenopathy and an unknown primary or tongue mass. If sufficient fine needle aspiration biopsy material is available, thyroid transcription factor-1, thyroglobulin, or molecular testing may prove useful in differentiating cribriform adenocarcinoma of salivary glands from papillary thyroid carcinoma. A misdiagnosis of papillary thyroid carcinoma may lead to inappropriate treatment including unnecessary thyroidectomy. Therefore, it is critical for both pathologists and surgeons to be aware of this uncommon entity to avoid misdiagnosis and subsequent mismanagement.

## Background

Polymorphous adenocarcinoma (PAC) is the third most common malignant salivary gland tumor, but most of the literature to date remains as case reports. Cribriform adenocarcinoma of salivary glands (CASG) is a rare subtype of PAC and was first described in 1999 by Michal *et al*., who reported eight cases of adenocarcinoma of the tongue that all resembled papillary thyroid carcinoma (PTC) histopathologically[1]. Interestingly, all eight patients had regional neck disease and, after 2–6 years of follow-up, all were alive with no recurrence after primary excision and a course of adjuvant radiation. At the time, this was called cribriform adenocarcinoma of the tongue, as it was hypothesized that this tumor arose from remnants of the thyroglossal duct (TGD). In summarizing the literature, the most frequently reported location was the tongue (76%), followed by soft palate (7%), tonsils (7%), and retromolar mucosa (5%)[2]. Thus, the theory that this tumor arose from the tongue or TGD remnant was challenged, and the name was changed to cribriform adenocarcinoma of salivary glands. Our case adds to the small body of literature on this rare malignancy that has only 50–60 cases reported worldwide[3, 4].

## Case report

A 64-year-old Caucasian woman presented to a community otolaryngologist with a 4-year history of progressive postnasal drip and globus sensation. Her past medical history was significant for osteoarthritis, gastroesophageal reflux disease, and seasonal allergies. Her only medication was a fluticasone 50 mcg/dose nasal spray that she used once daily as needed during allergy season and famotidine 20 mg orally twice daily for gastroesophageal reflux disease. She worked as a jeweler and lived with a roommate. She had never been pregnant. There was no family history significant for cancer. She was a nonsmoker and did not consume alcohol. At her initial otolaryngologist appointment, she was diagnosed with laryngopharyngeal reflux (LPR). She was treated with pantoprazole magnesium 40 mg orally for 3 months. Despite initial treatment for LPR, these symptoms progressed and over the course of a year, she also developed dysphonia. Therefore, she sought a second opinion with a local otolaryngologist. She did not report any constitutional or infectious symptoms.

**Fig. 1:**
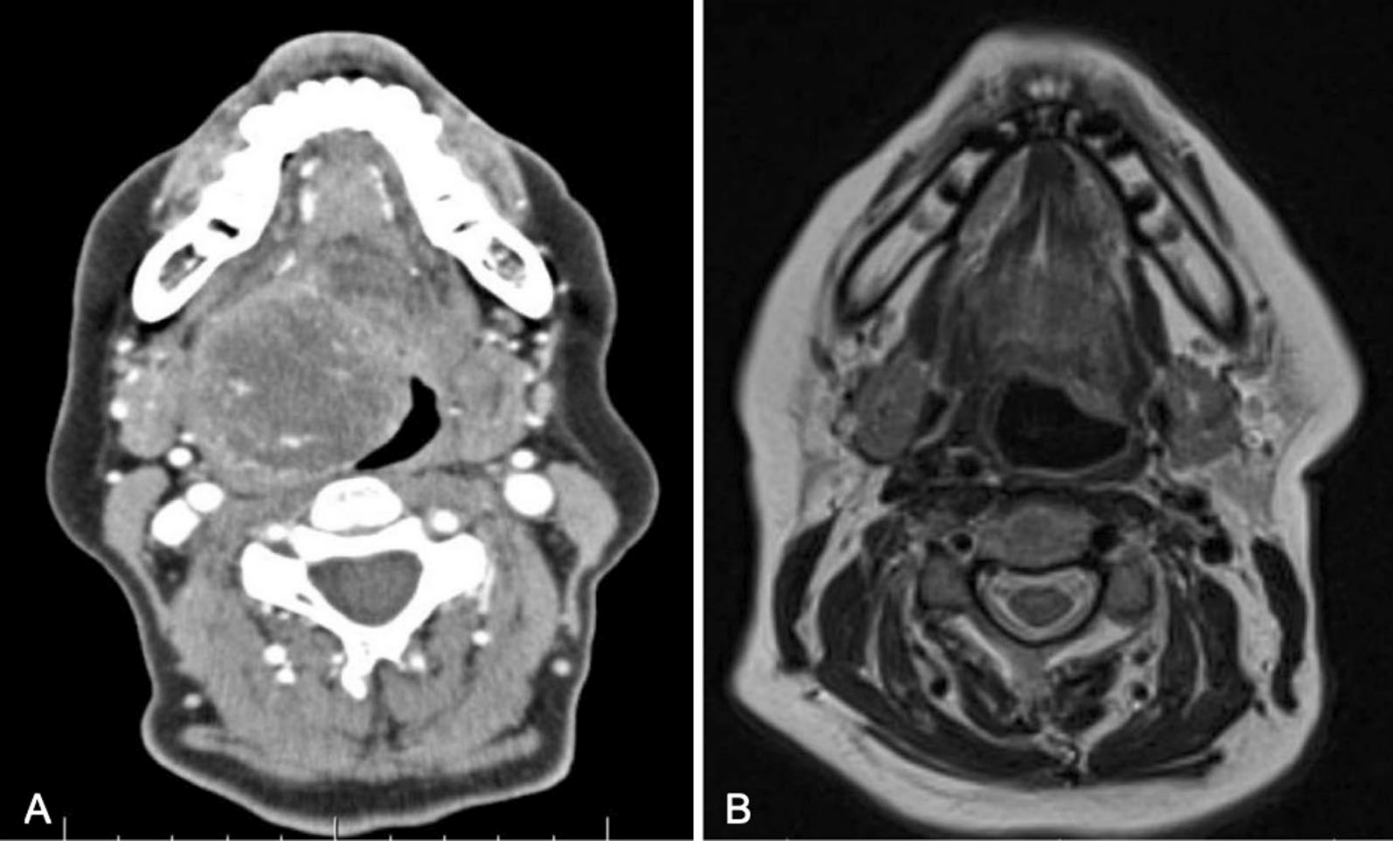
**A** (left) Preoperative CT imaging of tumor in axial section. **B** (right) Postoperative magnetic resonance imaging (MRI) in axial section

**Fig. 2:**
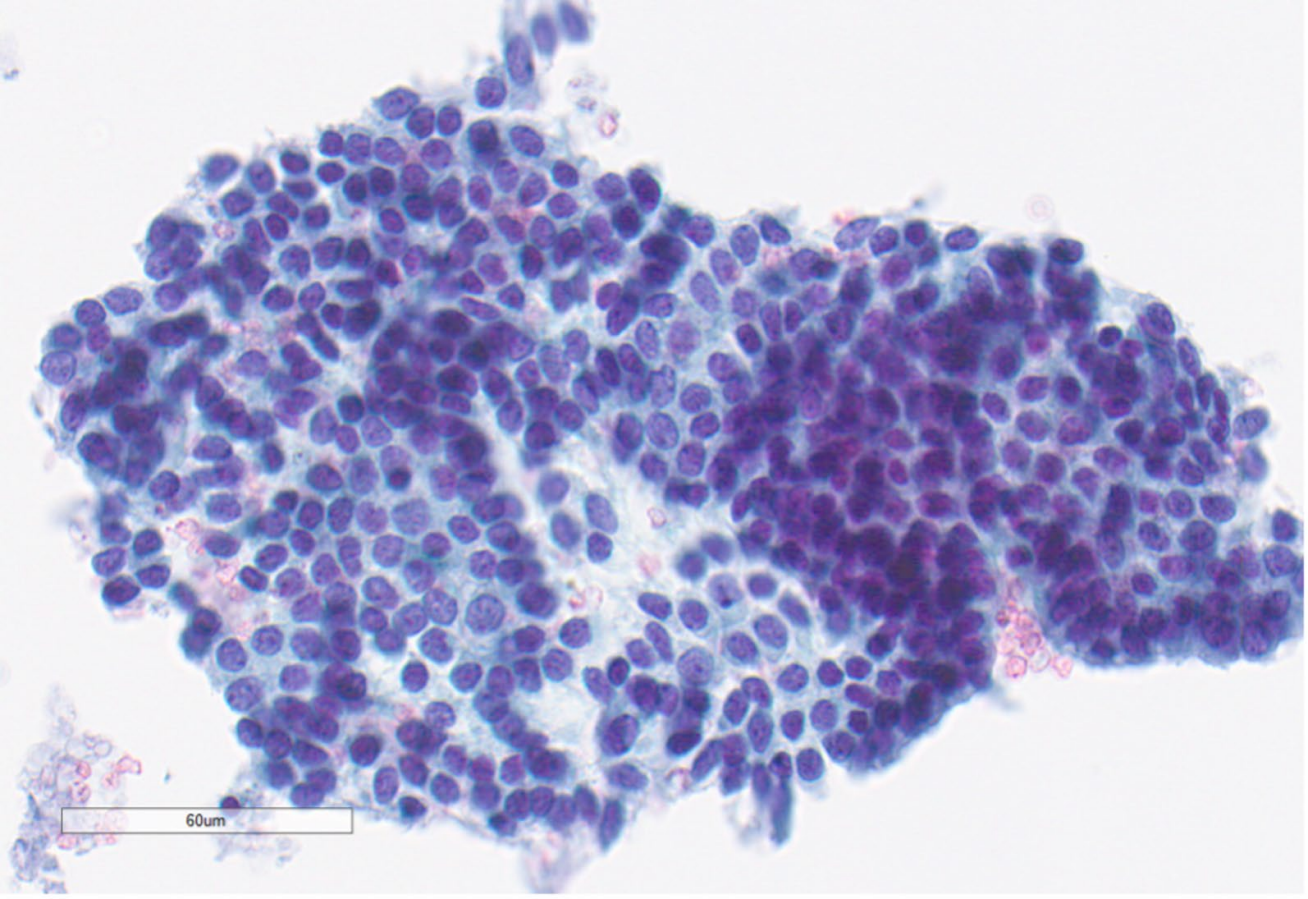
Pre-surgical fine needle aspiration biopsy showing papillary thyroid carcinoma-like nuclei with fine chromatin, ovality, nuclear grooves, and notching of nuclear membrane (×400)

On physical examination, she appeared clinically well. The oral cavity and oropharynx were normal and there was no palpable cervical lymphadenopathy. Her cranial nerves were intact and she was deemed to be a Glasgow Coma Scale of 15. The initial evaluation with flexible fiberoptic laryngoscopy showed a large vallecular lesion that was smooth and expansile, filling most of the oropharynx. It was, however, possible to pass the endoscope around the mass to visualize the glottis. The vocal cords appeared normal bilaterally. A neck ultrasound showed a well-defined hypoechoic mass with peripheral rim calcification located deep to the thyrohyoid membrane. The thyroid gland was found in a normal anatomic position and was without any nodules. Computed tomography (CT) imaging of the neck showed a rounded heterogeneous mass centered within the right aspect of the oropharynx measuring 4.2 × 4.4 × 4.5 cm and caused mass effect to the adjacent base of tongue, soft palate, and supraglottic larynx (Fig. 1A, B). Radiography did not reveal any suspicious neck adenopathy. Transcervical ultrasound-guided fine needle aspiration (FNA) biopsy was performed, which was suspicious for papillary carcinoma due to microscopic findings of malignant cells, nuclear grooves, and a powdery chromatin pattern (Fig. 2). Given the suspicion for papillary thyroid cancer (PTC), the case was discussed at an endocrine multidisciplinary case conference with recommendation for a staged surgical procedure. The first stage would be resection of the primary tumor, followed by a second-stage total thyroidectomy depending on the final pathology findings. The indication for a total thyroidectomy was size greater than 4 cm, in accordance with American Thyroid Association guidelines[5].

On the day of surgery, blood pressure measured 129/73 mmHg, pulse was 96 beats per minute, oxygen saturation was 100% on room air, and the patient was afebrile with a temperature of 37.1 °C. In the operating room, a tracheotomy was first performed for airway protection and the tumor was subsequently accessed through a transcervical, lateral pharyngotomy approach. The tumor was well encapsulated and involved the right base of tongue, lateral pharyngeal wall, and hyoid without gross invasion. The tumor was resected *en bloc* along with the right lateral hyoid, and the final specimen measured 3.6 × 3.8 × 5.9 cm. Of note, a limited cervical lymphadenectomy was also performed, primarily for access to the lateral pharynx, and two of three lymph nodes showed regional metastatic disease. The postoperative recovery period was uncomplicated and she recovered well with no dysphagia or dysphonia. Overall, the patient was admitted for three nights and was discharged on postoperative day (POD) 3. During her hospital stay, she received 24 hours of intravenous cefazolin 1 g every 8 hours and metronidazole 500 mg every 12 hours. She received one dose of hydromorphone 1 mg on POD 0 in the evening for pain control. She received three doses of lorazepam 0.5 mg sublingual for anxiety, five total doses of acetaminophen 1 g for pain, chlorhexidine 0.12% 15 ml mouth rinses every 6 hours, and enoxaparin 40 mg subcutaneously every night for the three nights of her admission. Acetaminophen was given via nasogastric tube for the first 2 days, then orally when she was tolerating a fluid diet. She was started on clear fluids and advanced to full fluids on POD 2, then puree diet on POD 3. She was seen by a speech language pathologist on POD 3 who deemed her safe to swallow orally. Her tracheostomy tube and surgical drain were removed on POD 3. Her bloodwork was unremarkable: hemoglobin 124 g/L, white blood cell count 10.9 × 10^9^/L, platelets 323 × 10^9^/L, sodium 140 mmol/L, potassium 3.8 mmol/L, creatinine 54 μmol/L, lactate 1.0 mmol/L, bilirubin 3 μmol/L, gamma-glutamyl transferase 6 IU/L, alkaline phosphatase 120 IU/L, alanine transaminase 15 IU/L, and lipase 125 IU/L. Her coronavirus disease 2019 (COVID-19) test was negative. Upon discharge, she was prescribed acetaminophen 1 g orally every 6 hours as needed for 2 weeks, chlorhexidine 0.12% 15 ml mouth rinses every 6 hours for 2 weeks, and hydromorphone 1 mg orally every 4 hours as needed (15 tabs).

**Fig. 3:**
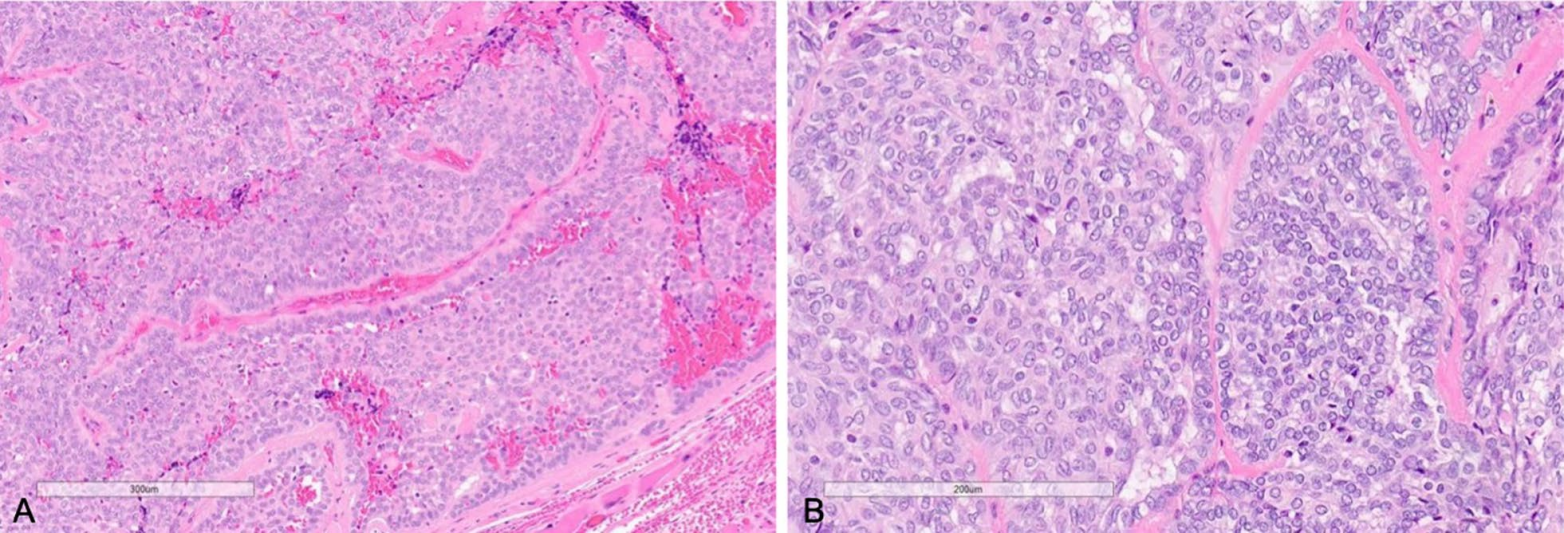
**A** (left) Histological analysis of resected tumor showing solid growth pattern with peripheral palisading and intervening fibrous stroma (×400). **B** (right) Polymorphous adenocarcinoma, cribriform type (cribriform adenocarcinoma of salivary glands, CASG) tumor cells with open-to-clear chromatin, ovality, and nuclear grooves, resembling the nuclei of papillary thyroid carcinoma (×400)

On microscopy, the tumor was composed of lobules of cytologically uniform cells forming cribriform, microcystic, solid, and tubular growth patterns (Fig. 3). Peripheral palisading was present in the solid areas. Cleft formation, glomeruloid, and occasional papillary structures were seen. The intervening stroma was fibrovascular with focal hyalinization and mucoid stroma. The mass had a rounded edge with focal irregular infiltration into adjacent muscle. The tumor nuclei were uniform, round to oval with fine chromatin and inconspicuous nucleoli. Nuclear grooves, nuclear membrane notching, and occasional intranuclear pseudoinclusions were identified. Many nuclei showed optical clearing. Immunohistochemistry was positive for CK7, S100, SOX-10, and p63 (focal, patchy), and negative for thyroglobulin, thyroid transcription factor-1 (TTF-1), p40, and GFAP. Molecular testing by RNA fusion panel revealed a CTNNB1-PRKD2 rearrangement. Overall, this was classified as a low-grade adenocarcinoma and staged as pT3 pN2b.

After a discussion regarding her case at a local multidisciplinary tumor board, options for close surveillance versus adjuvant radiation were presented to the patient at a 1-month follow-up. She declined radiation after extensive discussion and opted for surveillance. She underwent a flexible nasolaryngoscopy at that time, which showed a healed base of tongue, no evidence of disease recurrence, and no palpable lymphadenopathy. She underwent a follow-up magnetic resonance imaging (MRI) of her neck, which was normal and did not show any lymphadenopathy or tumor regrowth. She was then seen again in follow-up at 6 months and 10 months postoperatively. At both visits, she was clinically well with no symptoms of dysphagia or dysphonia. Her flexible nasolaryngoscopy did not show any disease recurrence; clinical palpation of her neck did not reveal any lymphadenopathy and ultrasound evaluation of her neck confirmed this. She is currently scheduled for yearly restaging imaging, which she will undergo in the coming months.

## Discussion

Overall, this was the case of a 64-year-old otherwise healthy woman who presented with a large base of tongue lesion. Initial FNA biopsy showed overlapping characteristics of papillary thyroid cancer (PTC) and CASG. After a multidisciplinary tumor board discussion, decision was made to proceed with primary resection and possibility for a second-stage thyroidectomy depending on pathology. Final histopathology confirmed CASG. The patient declined adjuvant radiation and was clinically disease free at 10 months postoperatively. Our case report describes the largest CASG reported thus far in literature with the longest follow-up time without adjuvant treatment. It highlights both the clinical and histological differences between PTC and CASG, framing it in a clinically relevant manner to surgeons and pathologists alike[2, 6, 7].

Diagnostically, CASG is certainly challenging as initial presentation and cytologic nuclear features can be easily confused with PTC arising from a TGD remnant or lingual thyroid. On FNA cytology, CASG shows tumor cells with elongated, overlapping nuclei with finely granular chromatin, punctate nucleoli, and moderate amounts of soft, fluffy cytoplasm[2]. This is nearly identical to PTC that has irregular nuclear contours, nuclear grooves, and rare intranuclear inclusions. In 2014, Gailey *et al*. reported that a key distinguishing feature is the “salivary gland-type” background of CASG that has fragments of metachromatic stroma and flocculent pink myxoid/mucoid material that a pathologist must differentiate from colloid[2]. Moreover, TTF-1 and thyroglobulin tests are negative for CASG but positive for PTC. By immunohistochemistry, it has been reported that this tumor reacts with AE1/AE3, CAM5.2, CK7, and CK8/18 as well as S-100 protein, p63, CK5/6 (variable), CK14, calponin, and smooth muscle actin[8]. The majority of CASGs have been shown to have PRKD2 fusions/rearrangements rather than point mutations, the latter of which are more common in PAC. Clinically, CASG also has significantly higher rates of nodal metastasis and histological differences when compared with PAC [3]. These differences have introduced controversy into the classification of CASG as a subtype of PAC or as a wholly separate diagnostic entity. In 2013, there were only 31 cases described worldwide of CASG, of which 67.7% occurred in the base of tongue and the majority of patients presented with neck metastases, echoing the findings in this study [8–13]. Recent retrospective studies, the largest of which was made up of 69 patients from 1987–2015, have also compared and contrasted PAC and CASG and advocated for CASG as a separate entity given its histopathological differences and rarity compared with PAC[3]. In the last decade following this review, CASG has also been described in several case reports arising in more uncommon places, such as the nasopharynx or parotid gland, but few have provided an in-depth characterization of the diagnostic pitfalls between CASG and PTC which is the most common challenge due to location of tumor[14–16]. If sufficient FNA biopsy material is available, TTF-1, thyroglobulin, or molecular testing may prove useful in differentiating CASG from PTC and PAC. Treatment with surgical excision with or without adjuvant radiation have both been described as appropriate management. Though this case report highlights the key pathological and clinical characteristics of CASG in extensive detail, a larger study cohort or systematic review of worldwide cases would provide better context for this rare disease.

## Conclusion

It is difficult to distinguish CASG from PTC solely by cytology, and the distinct characteristics of regional lymph node metastasis coupled with nuanced histologic differences should be emphasized in the evaluation of patients presenting with neck lymphadenopathy and an unknown primary or tongue mass. A misdiagnosis of PTC may lead to inappropriate treatment including unnecessary thyroidectomy. Therefore, it is critical for both pathologists and surgeons to be aware of this uncommon entity to avoid misdiagnosis and subsequent mismanagement.

## Data Availability

The datasets used and/or analyzed during the current study are available from the corresponding author on reasonable request.

## Abbreviations

PAC: Polymorphous adenocarcinoma
CASG: Cribriform adenocarcinoma of salivary glands
PTC: Papillary thyroid cancer
TGD: Thyroglossal duct
LPR: Laryngopharyngeal reflux
CT: Computed tomography
FNA: Fine needle aspiration
